# Electroacupuncture Treatment Alleviates the Remifentanil-Induced Hyperalgesia by Regulating the Activities of the Ventral Posterior Lateral Nucleus of the Thalamus Neurons in Rats

**DOI:** 10.1155/2018/6109723

**Published:** 2018-11-11

**Authors:** Hong-Yan Zhao, Ling-Yu Liu, Jie Cai, Yan-Jun Cui, Guo-Gang Xing

**Affiliations:** ^1^Neuroscience Research Institute and Department of Neurobiology, School of Basic Medical Sciences, Peking University, Beijing 100083, China; ^2^Department of Internal Medicine, Peking University Hospital, Beijing 100871, China; ^3^The Second Affiliated Hospital of Xinxiang Medical University, Xinxiang, Henan 453002, China; ^4^Key Laboratory for Neuroscience, Ministry of Education/National Health and Family Planning Commission, Beijing 100083, China

## Abstract

Mechanisms underlying remifentanil- (RF-) induced hyperalgesia, a phenomenon that is generally named as opioid-induced hyperalgesia (OIH), still remain elusive. The ventral posterior lateral nucleus (VPL) of the thalamus, a key relay station for the transmission of nociceptive information to the cerebral cortex, is activated by RF infusion. Electroacupuncture (EA) is an effective method for the treatment of pain. This study aimed to explore the role of VPL in the development of OIH and the effect of EA treatment on OIH in rats. RF was administered to rats via the tail vein for OIH induction. Paw withdrawal threshold (PWT) in response to mechanical stimuli and paw withdrawal latency (PWL) to thermal stimulation were tested in rats for the assessment of mechanical allodynia and thermal hyperalgesia, respectively. Spontaneous neuronal activity and local field potential (LFP) in VPL were recorded in freely moving rats using the *in vivo* multichannel recording technique. EA at 2 Hz frequency (pulse width 0.6 ms, 1–3 mA) was applied to the bilateral acupoints “Zusanli” (ST.36) and “Sanyinjiao” (SP.6) in rats. The results showed that both the PWT and PWL were significantly decreased after RF infusion to rats. Meanwhile, both the spontaneous neuronal firing rate and the theta band oscillation in VPL LFP were increased on day 3 post-RF infusion, indicating that the VPL may promote the development of RF-induced hyperalgesia by regulating the pain-related cortical activity. Moreover, 2 Hz-EA reversed the RF-induced decrease both in PWT and PWL of rats and also abrogated the RF-induced augmentation of the spontaneous neuronal activity and the power spectral density (PSD) of the theta band oscillation in VPL LFP. These results suggested that 2 Hz-EA attenuates the remifentanil-induced hyperalgesia via reducing the excitability of VPL neurons and the low-frequency (theta band) oscillation in VPL LFP.

## 1. Introduction

As an ideal ultrashort-acting opioid, remifentanil (RF) is widely used in clinical anesthesia. But after long-time or high-dose infusion, patients tend to appear hyperalgesia after RF withdrawal, a phenomenon that is generally named as opioid-induced hyperalgesia (OIH). The clinical symptoms of OIH are manifested as an increased sensitivity to noxious stimuli, and preclinical experiments have shown that mechanical and thermal hyperalgesia appeared after RF withdrawal [1–3]. How to prevent and treat the remifentanil-induced hyperalgesia and subsequently to investigate its underlying mechanism has become a research hotspot in recent years.

The ventral posterior lateral nucleus (VPL) of the thalamus is an important relay station for the transmission of nociceptive information to the cerebral cortex and sends up a sensory aspect of nociceptive information to the primary somatosensory cortex (SI) and other cortical areas for further processing [4]. In patients with neuropathic pain, the development of pain and pain modulation are related to the power of oscillations (e.g., theta, alpha, and beta oscillations) in local field potentials (LFPs) [5]. In animal models of neuropathic pain, increased spontaneous discharges, enlarged pain receptive fields, and rhythmic oscillatory firing in VPL neurons are observed [6–8]. In rats with central pain after spinal cord injury, neurons in the VPL firing spontaneously at a high rate, with increased after-discharges and evoked responses [9]. Both preclinical and clinical studies have found that VPL and other brain regions can be activated by RF [10–12]. But there has been less evidence for explaining the role of VPL in OIH. We thus first investigated the role of VPL in the development of OIH by *in vivo* multichannel recording in this study.

Electroacupuncture (EA), a type of acupuncture with electrical stimulation, is a safe and effective method for the treatment of pain and is widely applied in clinical settings [13–15]. Neuroimaging studies have shown that acupuncture or EA applications elicit extensive changes in multiple brain regions [16, 17], which overlapped with most of the neural networks of pain transmission and perception, including the spinal cord, the thalamus, and the cerebral cortex [18]. In addition, EA treatment also inhibits the activity of nociceptive neurons in the VPL [19], and 2 Hz-EA relieves the remifentanil-induced hyperalgesia in rats [20]. We therefore speculated that 2 Hz-EA might alleviate OIH by altering the activity of VPL neurons in rats.

In the present study, we aimed to explore the role and the underlying mechanisms of VPL in the development of RF-induced hyperalgesia in rats by using the *in vivo* multichannel recording technique. Furthermore, we investigated whether EA treatment could alleviate the RF-induced hyperalgesia by regulating the activity of VPL neurons in rats. We found that both the excitability of VPL neurons and the activity of theta band power spectral density (PSD) in VPL LFP are increased after RF infusion, indicating that the VPL may promote the development of RF-induced hyperalgesia by regulating the pain-related cortical activity. Moreover, we demonstrated that 2 Hz-EA attenuates the remifentanil-induced hyperalgesia via reducing the excitability of VPL neurons and the activity of low-frequency (theta band) oscillation in VPL LFP.

## 2. Materials and Methods

### 2.1. Animals

Male Sprague-Dawley rats weighing 180~220 g at the beginning of the experiment were provided by the Department of Experimental Animal Sciences, Peking University Health Science Center. The rats were kept in separated cages, free to get food and water, and kept in the animal feeding box controlled by temperature (24 ± 1°C), humidity (50~55%), and irradiation (12 : 12 h light : dark cycle). All animal experimental procedures were carried out in accordance with the guidelines of the International Association for the Study of Pain [21] and were approved by the Animal Care and Use Committee of Peking University (No. LA2016060, 2016). The behavioural experimenters were kept blind from the groupings of the rats.

### 2.2. Drug Administration

A 24-gauge disposable catheter (24 G, flow: 23 ml/min, *L* = 20 mm, Shanghai Puyi Medical Instruments Co. Ltd.) was flushed with heparinized saline and inserted into the caudal vein. The caudal vein catheter was used to infuse normal saline (Datong Huida Pharmaceutical Co. Ltd.) and Ruijie® (remifentanil hydrochloride for injection, H20030197, Yichang Humanwell Pharmaceutical Co. Ltd., China) dissolved in normal saline. Rats were anesthetized by pentobarbital sodium (50 mg·kg^−1^, i.p.). The rats were assigned randomly to a treatment group that received remifentanil (RF) intravenously or to a control group that received normal saline (vehicle) via the tail vein. The treatment protocol was performed as follows: rats in RF group received remifentanil infusion (1.5 *μ*g·kg^−1^·min^−1^), while rats in vehicle group rats received 0.9% normal saline infusion (0.1 ml·kg^−1^·min^−1^). The infusion lasted for 2 hours, and spontaneous breaths and eyelash reflexes were monitored to ensure the rats' wellbeing.

## 3. Behavioural Tests

### 3.1. Assessment of Mechanical Allodynia

Mechanical allodynia was assessed by measuring 50% paw withdrawal threshold (PWT) in rats as described in our previous reports [22–25]. In brief, the 50% PWT in response to a series of von Frey filaments (Stoelting, Wood Dale, IL) was determined by the up-and-down method [26]. Rats were placed on a metal mesh floor covered with an inverted clear plastic cage (18 × 8 × 8 cm) and allowed for a habituation period of 20 minutes. Eight von Frey filaments (0.4, 0.7, 1.2, 2.0, 3.6, 5.5, 8.5, and 15.1 g) with approximately equal logarithmic increments (0.224) of bending force were selected. Each test began with a delivery of a 2.0 g von Frey filament force perpendicular to the plantar surface of the hind paw for approximately 2~3 s. An abrupt withdrawal of the foot immediately after stimulation or immediately after removal of the filament was recorded as a positive response. Whenever there was a positive or negative response, the next weaker or stronger filament was applied, respectively. This procedure was completed until the six stimuli after the first change in response were observed. The 50% PWT was calculated using

(1) $$\begin{array}{c} \;50\%PWT=10^{\left[X_{f}+\kappa \delta \right]}, \end{array}$$

where *X*_f_ is the value of the final von Frey filament used (in log units), *κ* is a value measured from the pattern of positive/negative responses, and *δ* = 0.224, which is the average interval (in log units) between the von Frey filaments [27]. If an animal responded to the lowest filament, a value of 0.25 g was assigned. If an animal did not respond to the highest filament, the value was recorded as 15.0 g. The allodynic rats were defined as 50% PWT which is less than 4.0 g (i.e., withdrawal in response to innoxious tactile stimulation). Testing sessions were performed on day 1 prior to RF infusion as well as on days 1, 3, 5, 7, 9, and 11 post-RF infusion.

### 3.2. Assessment of Thermal Hyperalgesia

Thermal hyperalgesia of the hind paws was tested according to our previous studies [23, 25, 28, 29]. Briefly, rats were adapted to the acrylic enclosures on a clear glass plate maintained at 30°C for at least 30 minutes. A radiant heat source was focused onto the plantar surface of the hind paw. Measurements of paw withdrawal latency (PWL) were taken by a timer that was started by the activation of the heat source and stopped when withdrawal of the paw was detected with a photo detector. A maximal cut-off time of 30 seconds was applied to avoid unnecessary tissue damage. Three measurements of PWL to each hind paw were taken and averaged as the result of each test session. The hind paw was alternately tested between consecutive tests with intervals greater than 5 minutes. Testing sessions were performed on day 1 prior to RF infusion as well as on days 1, 3, 5, 7, 9, and 11 post-RF infusion.

### 3.3. Electroacupuncture (EA) Application

Rats were restrained in rodent holders with their hind legs and tails protruding [30]. Briefly, a pair of stainless steel needles (0.2 mm in diameter, 5 mm in length) was inserted into acupoints “Zusanli” (ST.36, 4 mm lateral to the anterior tubercle of the tibia, which is marked by a notch) and “Sanyinjiao” (SP.6, 3 mm proximal to the medial malleolus, at the posterior border of the tibia). Electrical stimulation of a constant current generated from Han's Acupoint Nerve Stimulator (HANS, LH202H, Beijing Astronautics and Aeronautics Aviation University, Beijing, China) was delivered to bilateral hind limbs simultaneously. This electrical stimulation was set as a square wave of 0.6 ms in pulse width and 2 Hz in frequency. The electrical pulses were delivered from 1 to 3 mA in a step of 1 mA increment. Each step of the stimulus lasted for 10 min. The EA stimulation was given for 30 min on day 1 prior to RF infusion, continued during the 2-hour RF infusion, and three times (once per day) on days 1, 2, and 3 after RF infusion. Animals allocated into the mock EA groups were then placed in the same apparatus and underwent needle insertion in the same acupoints, but no electrical current was applied to them. EA procedures were always carried out by the same experimenter.

## 4. *In Vivo* Multichannel Recording

### 4.1. Surgery

**Figure 1:**
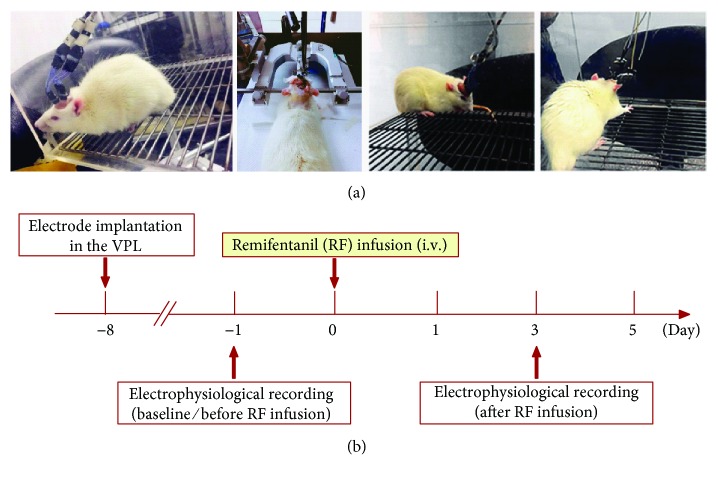
*In vivo* multichannel recording. (a) Surgery for the electrode implantation and the *in vivo* multichannel recording in freely moving rats. (b) Experimental procedure of *in vivo* multichannel recording. Data was collected on day 1 before and day 3 after remifentanil infusion.

Initial anesthesia was performed on the rats by sodium pentobarbital injection (50 mg/kg, i.p.) prior to implantation of the microelectrode array. A supplemental dose (1/3 of the original dose) of pentobarbital sodium was administered to the animals as needed to maintain proper anesthetic depth during surgery. Rats were mounted on a stereotaxic device (Reward Life Science Technology Co. Ltd., Shenzhen), and an array of eight nickel chromium alloy wire PEG 2000-insulated microwires (14 *μ*m in diameter, arranged in a 4 × 2 configuration, 250 *μ*m spacing between each microwire, STABLOHM 675, California Fine Wire Company, USA) was slowly lowered into the right VPL (−3.0 mm AP, −3.0 mm ML, and −6.0 mm DV) according to the rat atlas of Paxinos and Watson [31]. Six stainless steel screws were driven into the skull to serve as anchors for holding the microwires in place after implantation. Prior to surgery, rats received penicillin injection (16,000 IU, i.m.) to prevent infection, and then the animals were allowed to recover for one week before the start of recording (see Figure 1).

### 4.2. Electrophysiological Recording

*In vivo* multichannel recording was performed as described elsewhere [32]. Briefly, rats were placed in a transparent plastic chamber (44 × 44 × 44 cm) in a quiet room at 22 ± 1°C and allowed to move freely throughout the recording period. Electrophysiological signals were recorded after the animals adapted to the experimental environment. Data were collected at baseline (before infusion) and on day 3 after RF infusion (see Figure 1(b)). Multiunit neuronal activities were collected through the implanted microwire assemblies that were connected to a preamplifier via a headstage plug and a lightweight cable, and the ground wire was used as a reference. The outputs of the preamplifier were filtered (0.5 and 5 kHz, 6 dB cut-off) and sent to a multichannel spike-sorting device (Plexon Inc.) for online signal processing. Local field potentials (LFPs) were recorded from the right VPL by microwire arrays with a multichannel data acquisition system (Plexon Inc.). The LFP signals were transmitted from the headstage assemblies to the preamplifier via a lightweight cable. LFPs were collected at a sampling frequency of 10 kHz, amplified (300x), and band-pass filtered (0.3–500 Hz). LFP signals were filtered into four frequency bands: theta (4–8 Hz), alpha (9–12 Hz), beta (13–30 Hz), and gamma (31–100 Hz), in which the theta, alpha, and beta bands belong to the low-frequency band (4–30 Hz), while the gamma band belongs to the high-frequency band (31–100 Hz). In a transparent plastic chamber, LFP oscillations and the video recording for the animals' behaviours were simultaneously recorded during a 10-minute resting state. Spike train activity was analysed by NeuroExplorer (Plexon Inc.). Waveform capture and frequency distribution histogram were used to verify the classification of a single unit. Different waveforms were individually distinguished by setting multiple time-voltage windows using Offline Sorter software (Plexon Inc.). The time stamps of these waveforms were then stored on a personal computer for off-line analysis.

### 4.3. Data Analysis

According to a previous report [32], the neuronal firing rate was quantified for each neuron to construct rate histograms with a time range from the beginning to 5 min after recording in different groups. In brief, the bin size used to calculate the histograms was 2 seconds. Bin counts for each trial were calculated using the analysis program NeuroExplorer (Plexon Inc.), and the results were exported to Matlab (MathWorks Inc.) in spreadsheet form. The firing rates for all neurons were normalized and ranked into a spreadsheet for further statistical analysis. Power spectral density (PSD) was analysed using the NeuroExplorer (Plexon Inc.) and Matlab toolbox Chronux (http://chronux.org). Local field potential (LFPs) data were resampled at 1 kHz and analysed separately for each 5-minute recording period. The 50 Hz frequency was removed from the digitized signal prior to any analysis with a frequency resolution of ~1 Hz. The calculated parameter values were set as follows: Params. fpass = [0–100] (frequency domain for analysis between 0 and 100 Hz); Params. fpass = [4–8] (frequency domain for analysis between 4 and 8 Hz). The time-varying power spectra was then calculated by fast Fourier transformation (FFT). Spectral units were normalized by raw power spectral densities. Power spectral analysis was carried out to calculate PSD.

### 4.4. Histology

**Figure 2:**
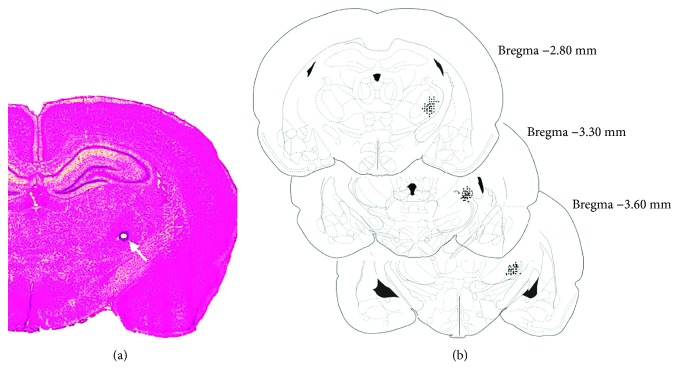
Histological verification of the recording electrodes sites in the right ventral posterior lateral nucleus (VPL) of the thalamus region. (a) Photomicrograph of a histological section stained with neutral red to illustrate the position of the recording electrode sites made by an electrolytic lesion through the respective electrodes (as arrow shows). (b) Representations of three coronal sections through the rat VPL are shown in sequence from anterior to posterior, respectively. The numbers in the right margin indicate millimeters posterior to the bregma. The filled circles in the right hemisphere show the approximate positions of the recording electrodes corresponding to some representative rats in the different groups.

After the termination of the experiment, the rats were deeply anesthetized with pentobarbital sodium and the tip positions of the electrodes were identified by a 2 mA, 10 s DC current (anode current) through the electrodes to produce a thermal lesion of the near area tissue. The animals were then sacrificed and perfused with 0.9% saline followed by 4% paraformaldehyde. After fixing in paraformaldehyde at 4°C overnight, the brains were transferred to a 20% sucrose solution in saline for cryoprotection. Coronal sections of 30 *μ*m were cut on a microtome, mounted on charged slides, and stained with neutral red dye for 10–15 min. Recording sites were determined under a light microscope (see Figure 2). Data of those sites deflecting from the target area were excluded from analysis.

### 4.5. Statistical Analysis

As described in previous reports [24, 33], statistical analyses were performed with GraphPad Prism 7.0 for Windows (GraphPad Software Inc., La Jolla, CA). All data were expressed as mean ± standard error of the mean. Two-way analysis of variance (ANOVA) followed by the Bonferroni post hoc test was used for multiple comparisons. Differences with *P* < 0.05 were considered statistically significant.

## 5. Results

### 5.1. Remifentanil Induces Mechanical Allodynia and Thermal Hyperalgesia in Rats

**Figure 3:**
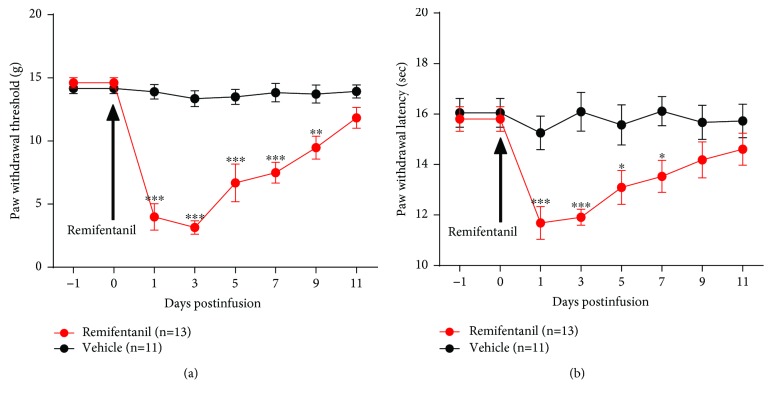
Effects of RF infusion on the paw withdrawal threshold (PWT) in response to mechanical stimuli and the paw withdrawal latency (PWL) to thermal stimulation in rats. (a) PWT. (b) PWL. Note that RF infusion results in a significant mechanical allodynia and thermal hyperalgesia as measured by a decreased PWT and PWL in rats. ^∗^*P* < 0.05, ^∗∗^*P* < 0.01, or ^∗∗∗^*P* < 0.001 versus the corresponding vehicle group, two-way ANOVA followed by the Bonferroni post hoc test, *n* = 11 vehicle and 13 RF.

Compared to the vehicle group, infusion of RF (1.5 *μ*g·kg^−1^·min^−1^ for 120 minutes) to rats induced a significant decrease both in the paw withdrawal threshold (PWT) in response to mechanical stimuli (on day 3 postinfusion, 13.3 ± 0.6 g vehicle vs. 3.1 ± 0.5 g RF, *P* < 0.0001, two-way ANOVA, *n* = 11 vehicle and 13 RF) (see Figure 3(a)) and the paw withdrawal latency (PWL) to thermal stimulation (on day 3 postinfusion, 16.1 ± 0.8 sec vehicle vs. 11.9 ± 0.3 sec RF, *P* < 0.0001, two-way ANOVA, *n* = 11 vehicle and 13 RF) (see Figure 3(b)). The results suggested that RF infusion resulted in a stable OIH in rats. Moreover, inclined-plate test was performed to assess the locomotor function of experimental animals. Expectedly, no significant motor dysfunction was observed in rats that received RF infusion (data not shown). In addition, we also performed an elevated plus-maze (EPM) test to determine whether RF infusion could produce an anxiety-like behaviour in rats. The results revealed that there were no significant differences in the time spent in the open arms as well as the number of entries in the open arms between the vehicle- and the RF-treated rats on day 3 after RF infusion (data not shown). These results suggested that RF infusion could not induce anxiety-like behaviour in rats.

### 5.2. Remifentanil Enhances the Spontaneous Neuronal Firing Rate and the Activity of Theta Band Oscillation in VPL LFP

The numbers of simultaneously recorded neurons were depicted as follows: 26 neurons pre- and 28 neurons postinfusion in the vehicle group and 30 neurons pre- and 30 neurons postinfusion in the RF group.

**Figure 4:**
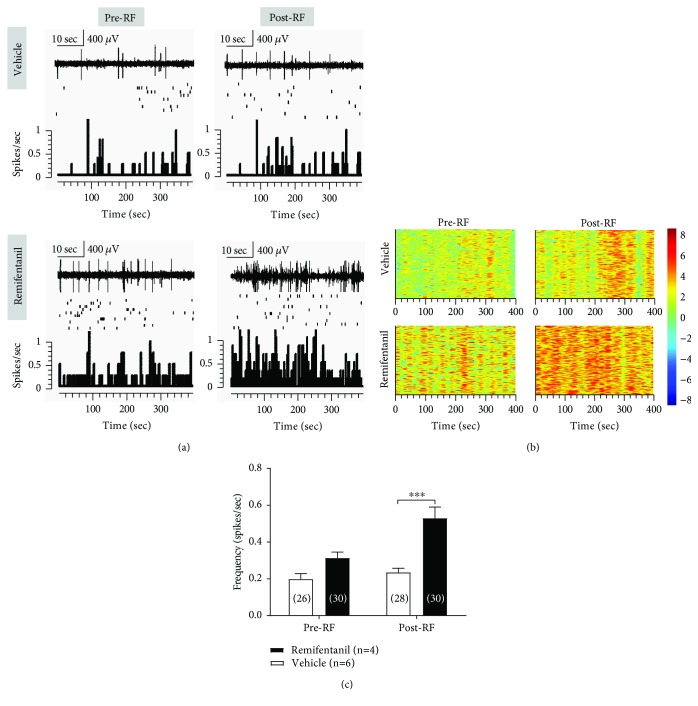
Effects of RF infusion on spontaneous neuronal activity in VPL neurons. (a) Representative spontaneous activity of VPL neurons before and after RF infusion in the vehicle- and RF-treated rats. Upper: representative traces of the neuronal discharges. Scale bar: 400 *μ*V, 10 sec. Middle: scatter plot of the spikes. Lower: histograms show the number of action potentials (spikes) per second (bin width, 2 seconds). (b) Cluster plots depicting activity of VPL neurons (rows) before and after RF infusion in the two groups are shown. Warm and cool colors indicate the increase and decrease, respectively, in the spikes of multichannel recorded neurons. (c) Averaged spontaneous activity of VPL neurons (spikes per second) before and after RF infusion in vehicle- and RF-treated rats. ^∗∗∗^*P* < 0.001 versus the corresponding vehicle group, two-way ANOVA followed by the Bonferroni post hoc test, *n* = 26 to 30 cells (four to six rats) per group.

To investigate the ongoing neuronal activity in the VPL associated with RF-induced hyperalgesia, we examined the changes in mean spontaneous firing rate of VPL neurons by *in vivo* multichannel recording in free-moving rats. Compared with the vehicle group, RF infusion enhanced the mean spontaneous firing rate of VPL neurons on day 3 postinfusion (spikes/s, 0.2 ± 0.02 vehicle vs. 0.5 ± 0.06 RF, *P* < 0.0001, two-way ANOVA, *n* = 26 to 30 cells (from four to six rats) per group; see Figures 4(a), 4(c)). Similar results were observed on the spontaneous firing density as depicted in the cluster plot within 400 seconds pre- and post-RF infusion in the vehicle- and RF-infusion groups, respectively (see Figure 4(b)). However, as compared with baseline, the vehicle group rats did not show significant changes in the mean spontaneous firing rate of VPL neurons on day 3 post-RF infusion (*P* > 0.05, two-way ANOVA, *n* = 26 to 30 cells (from four to six rats) per group; see Figure 4(a), 4(c)).

**Figure 5:**
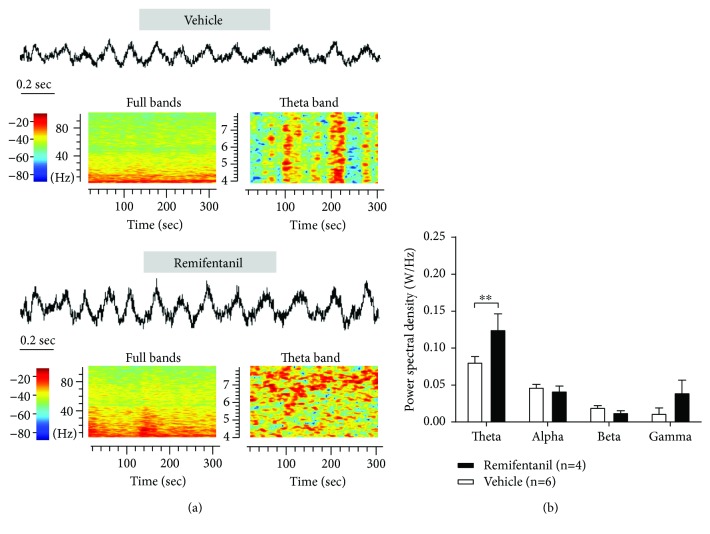
Effects of RF infusion on the activity of VPL LFP in rats. (a) An example of the LFP and the time-frequency representation in the vehicle- and RF-treated rats on day 3 post-RF infusion. Representatives of 4~100 Hz (left) and 4~8 Hz (right) frequency bands of LFP from the vehicle and RF groups are shown. The power spectra are normalized. Warm and cool colors indicate the increase and decrease, respectively, in field potential power of VPL LFP. (b) Summary of the power spectral density of theta, alpha, beta, and gamma bands on day 3 after RF infusion. Note that RF infusion induces a significant increase in activity of low-frequency theta (4–10 Hz) band but not alpha, beta, and gamma bands in VPL LFP. ^∗∗^*P* < 0.01 versus the corresponding vehicle group, two-way ANOVA followed by the Bonferroni post hoc test, *n* = 4 to 6 rats per group.

To further investigate the time-frequency relationship of theta, alpha, beta, and gamma oscillations of LFP in the VPL, we calculated the time-varying power spectra by FFT (see Figure 5(a)). The spectrum units were normalized by the raw power spectral density (PSD). Then, we performed power spectral analysis to compare the PSD of theta, alpha, beta, and gamma oscillations in the VPL LFP on day 3 post-RF infusion. The results showed that RF infusion induced a significant increase in the low-frequency theta (4–10 Hz) band activity of VPL LFP (W/Hz, 0.08 ± 0.01 vehicle vs. 0.13 ± 0.02 RF, *P* < 0.01, two-way ANOVA, *n* = 4 to 6 rats per group; see Figure 5(b)).

### 5.3. EA Treatment Attenuates the Remifentanil-Induced Mechanical Allodynia and Thermal Hyperalgesia in Rats

**Figure 6:**
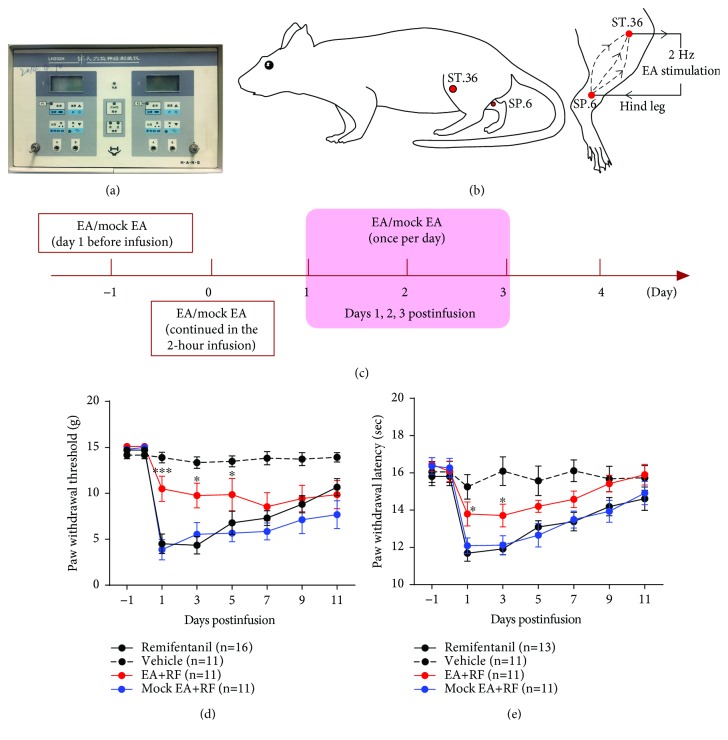
Effects of 2 Hz-EA treatment on the RF-induced hyperalgesia in rats. (a–b) Equipment and acupoints of EA stimulation ((a) Han's Acupoint Nerve Stimulator, LH202H; (b) a schematic diagram indicates the location of EA stimulation in rats). (c) Experimental procedure of the EA treatment. The EA stimulation was given for 30 min on the day preinfusion, continued in the 2-hour infusion, and three times (once per day) on days 1, 2, and 3 post-RF infusion. (d–e) Effects of EA treatment on the paw withdrawal threshold (PWT) in response to mechanical stimuli and the paw withdrawal latency (PWL) to thermal stimulation in rats that received RF infusion. Note that 2 Hz-EA treatment reverses the RF-induced decreases both in the PWT and PWL in rats. ^∗^*P* < 0.05 or ^∗∗∗^*P* < 0.001 versus the corresponding mock EA group, two-way ANOVA followed by the Bonferroni post hoc test, *n* = 11 to 16 rats per group.

Furthermore, we investigated the therapeutic effect of EA on OIH in a rat model of RF-induced hyperalgesia. The EA stimulation was performed by using Han's Acupoint Nerve Stimulator (HANS, LH202H) applied to the bilateral hind limbs of rats at the acupoints *Zusanli* (ST.36) and *Sanyinjiao* (SP.6) simultaneously (see Figures 6(a)–6(b)). The EA stimulation was given for 30 min per day before RF infusion, continued in the 2-hour infusion, and three times (once per day) on days 1, 2, and 3 post-RF infusion (see Figure 6(c)). The results showed that compared with the mock EA group, EA at 2 Hz frequency reversed the RF-induced decrease both in the PWT (on day 1 post-RF infusion, 10.5 ± 1.4 g EA vs. 3.9 ± 1.1 g mock EA, *P* < 0.0001; see Figure 6(d)) and in the PWL (on day 1 post-RF infusion, 13.8 ± 0.7 sec EA vs. 11.7 ± 0.4 sec RF, *P* < 0.05; see Figure 6(e)) in rats (two-way ANOVA, *n* = 11 to 16 rats per group). These results suggested that the 2 Hz-EA treatment could alleviate the RF-induced hyperalgesia in rats.

### 5.4. EA Treatment Abrogates the RF-Induced Augmentation of the Spontaneous Neuronal Activity and the PSD of Theta Band Oscillation in VPL LFP

The numbers of simultaneously recorded neurons were depicted as follows: 24 neurons pre- and 17 neurons postinfusion in the mock EA group and 20 neurons pre- and 16 neurons postinfusion in the EA group.

**Figure 7:**
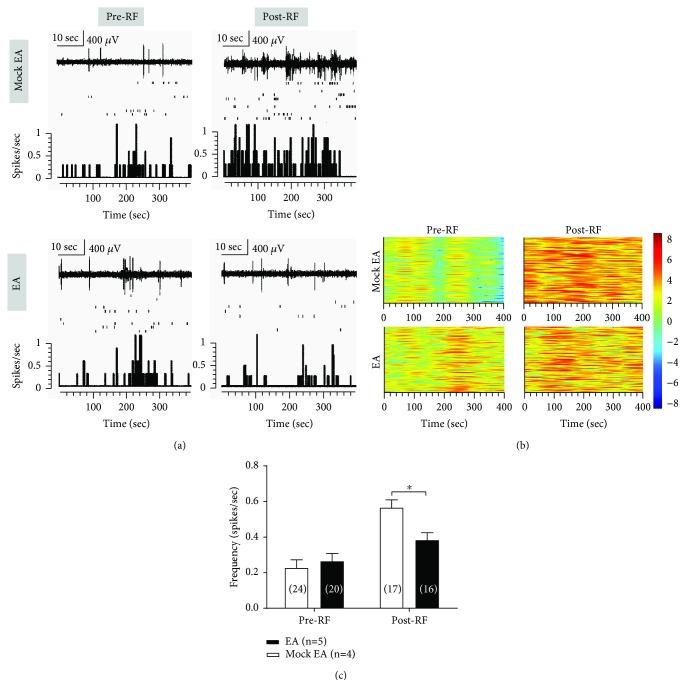
Effects of 2 Hz-EA treatment on the spontaneous neuronal activity of VPL neurons in rats that received RF infusion. (a) Representative spontaneous activity of VPL neurons before and after RF infusion in the EA- and mock EA-treated rats. Upper: representative traces of the neuronal discharges. Scale bar: 400 *μ*V, 10 sec. Middle: scatter plot of the spikes. Lower: histograms show the number of action potentials (spikes) per second (bin width, 2 seconds). (b) Cluster plot depicting activity of VPL neurons (rows) before and after RF infusion in the EA- and mock EA-treated rats are shown. Warm and cool colors indicate the increase and decrease, respectively, in the spikes of multichannel recorded neurons. (c) Averaged spontaneous activity of VPL neurons (spikes per second) before and after RF infusion in EA- and mock EA-treated rats. ^∗^*P* < 0.05 versus the corresponding mock EA group, two-way ANOVA followed by the Bonferroni post hoc test, *n* = 16 to 24 cells (four to five rats) per group.

Compared with the mock EA group, the 2 Hz-EA treatment could rescue the RF-induced increase in the spontaneous neuronal activity of VPL neurons on day 3 post-RF infusion (spikes/s, 0.4 ± 0.04 EA vs. 0.6 ± 0.04 mock EA *P* < 0.05, two-way ANOVA, *n* = 16 to 24 cells (from four to five rats) per group; see Figures 7(a), 7(c)). Similar results were observed on the spontaneous firing density as depicted in the cluster plot within 400 seconds pre-RF and post-RF infusion in the EA and mock EA groups (see Figure 7(b)).

**Figure 8:**
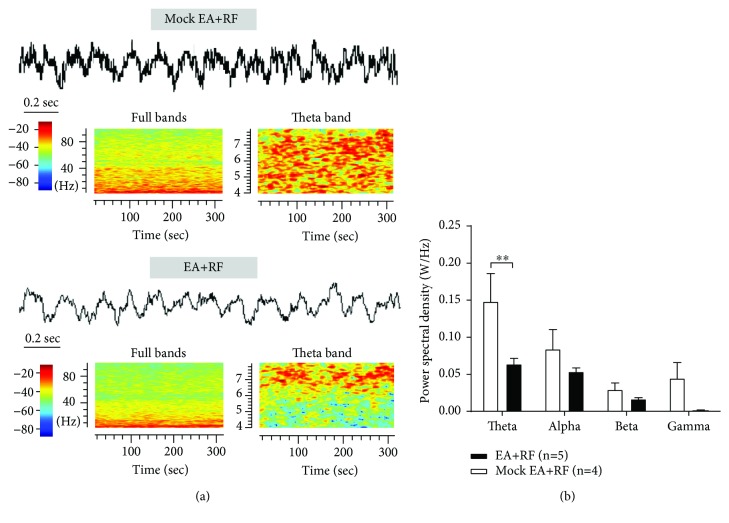
Effects of 2 Hz-EA treatment on the activity of VPL LFP in rats that received RF infusion. (a) An example of the LFP and the time-frequency representation in the EA- and mock EA-treated rats on day 3 post-RF infusion. Representatives of 4~100 Hz (left) and 4~8 Hz (right) frequency bands of LFP from the EA- and mock EA-treated rats are shown. The power spectra are normalized. Warm and cool colors indicate the increase and decrease, respectively, in field potential power of VPL LFP. (b) Summary of the power spectral density of theta, alpha, beta, and gamma bands on day 3 after RF infusion in the EA and mock EA groups. Note that 2 Hz-EA treatment abrogates the RF-induced increase in the power spectral density (PSD) of theta band oscillation (4–10 Hz) in rats on day 3 post-RF infusion. ^∗∗^*P* < 0.01 versus the corresponding mock EA group, two-way ANOVA followed by the Bonferroni post hoc test, *n* = 4 to 5 rats per group.

Through the power spectrum analysis of the time-frequency spectrogram in the LFP, we found that the 2 Hz-EA treatment also abrogated the RF-induced increase in the power spectral densities (PSD) of theta band oscillation (4–10 Hz) in VPL LFP (W/Hz, 0.06 ± 0.01 EA vs. 0.1 ± 0.04 mock EA, *P* < 0.01, two-way ANOVA, *n* = 4 to 5 rats per group; see Figure 8).

## 6. Discussion

### 6.1. Remifentanil-Induced Hyperalgesia in Rats

In order to study the underlying mechanisms of OIH, we first developed an animal model of RF-induced hyperalgesia in rats. In line with previous findings [10], we found that the RF-induced hyperalgesia was associated with the dose and duration of remifentanil infusion. Clinically, remifentanil is usually administered intravenously to patients at a dose of 0.1~0.3 *μ*g·kg^−1^·min^−1^. Since the equivalent dose in rats is 6.259 times of that in humans, the corresponding dose in rats is approximately 0.625~1.875 *μ*g·kg^−1^·min^−1^. A study of mice showed that the half effective doses (ED_50_) of RF-induced thermal and mechanical hyperalgesia were 1.7 *μ*g·kg^−1^·min^−1^ (95% confidence interval: 1.3~2.1) and 1.26 *μ*g·kg^−1^·min^−1^ (1.0~1.6), respectively [34]. Since a larger dose of RF can induce a more significant OIH phenomenon [35], we thus chose a dose of 1.5 *μ*g·kg^−1^·min^−1^ to develop a rat model of RF-induced hyperalgesia. A previous study has shown that the RF-induced hyperalgesia requires sufficient drug exposure time, and the duration of RF exposure is the main factor influencing the hyperalgesia induction [10]. Other studies also demonstrated that after 90 min of continuous infusion of RF, the area of mechanical hyperalgesia on the skin of rats is significantly enlarged, and more than 2 hour duration of infusion increases the incidence of RF-induced hyperalgesia [36, 37]. Based on our preliminary data, a long-term infusion (3 hours) of RF could result in severe respiratory depression, we thus developed a rat model of RF-induced hyperalgesia by infusing RF at 1.5 *μ*g·kg^−1^·min^−1^ for 2 hours in this study. In line with previous findings [1, 20], our present study revealed that after a 2-hour continuous infusion of RF, the rats showed significant mechanical allodynia and thermal hyperalgesia that lasted for almost one week. Moreover, we also observed that the postoperative hyperalgesia caused by RF infusion in a rat model of plantar incision could last for at least two weeks (data not shown), which far exceeded the recovery period of incisional pain only (within a week). Apart from the pain sensitivity, we also have tested the locomotor function and the anxiety-like behaviour in rats after RF infusion. We found that no significant motor dysfunction and anxiety-like behaviour appeared in rats on day 3 post-RF infusion. We speculated that it is possibly due to the time of our observation was relatively earlier after RF infusion; although the hyperalgesia was most severe on day 3 post-RF infusion, it may have not caused the pain-related negative emotions yet.

### 6.2. Remifentanil Enhances the Excitability of VPL Neurons in Rats

The thalamus is a gateway for the transmission of sensory information from the spinal cord to the cerebral cortex, where the VPL is projected to the primary somatosensory cortex (SI), secondary somatosensory cortex (SII), and other cortical areas for further processing. After the systematic infusion of RF in human volunteers, the functional magnetic resonance imaging (fMRI) results showed that the cerebral blood flow in the brain regions such as the anterior cingulate cortex (ACC) and the VPL was increased significantly, suggesting that these brain regions can be activated by RF infusion [38, 39]. In addition, systematic administration of morphine could increase the spontaneous firing rate of the VPL neurons [40]. Since the SI receive dense projections from the VPL, a major target for ascending sensory tracts in the spinal cord and brain stem, a large number of neurons in the SI are activated in a rat model of RF-induced hyperalgesia [41]. Our present results showed that the pain-related spontaneous firing rate of VPL neurons was increased in RF-treated rats. These data indicated that the increased excitability of VPL neurons may lead to a hyperexcitability in the pain-related brain regions such as SI and SII, thereby promoting the development of RF-induced hyperalgesia. In fact, evidence from human studies has revealed that the thalamocortical dysrhythmia and altered thalamic burst firing play a vital role in neuropathic pain [42, 43]. In patients with neuropathic pain, the thalamic neurons fire low-threshold calcium spike (LTS) bursts, which are thought to be mediated by T-type calcium channels [44]. These abnormal bursts are critical for the generation of theta rhythms during sleep [45, 46] as well as delta and theta oscillations in thalamic LFPs [44]. Hence, the LTS bursts of thalamic relay neurons exert a rhythmic influence on thalamocortical modules in the theta frequency band, which may underlie the pathophysiology of RF-induced hyperalgesia or chronic neuropathic pain [47].

### 6.3. Remifentanil Increases the Activity of Theta frequency Oscillation in VPL LFP

Brain rhythmical oscillations in the low (delta, theta, and alpha) and high (beta and gamma) frequencies of electroencephalography are linked to broad varieties of perceptual, sensorimotor, and cognitive operations [48]. For example, oscillatory brain activities in the high-frequency gamma band play an important role in selecting and integrating sensory-relevant information into a coherent perception [49]. Accumulative evidence has demonstrated that gamma band oscillations also participate in pain perception [50–52] and the abnormal working memory of chronic pain [53]. An increased gamma band oscillatory activity was also observed in the frontal-central region in subjects suffering from tonic muscle pain [52] or tonic heat pain [54]. The beta band oscillation may be involved in the maintenance of the current sensorimotor or cognitive state, and the extraordinary enhancement of the beta oscillation may result in an abnormal persistence of the current situation and a deterioration of flexible behaviours and cognitive controls [55]. With respect to the low-frequency oscillations, the delta band oscillation is associated with compromised neuronal function [56], the alpha band oscillation mainly serves as a top-down controlled inhibitory mechanism [57], and the theta band oscillation promotes neural plasticity [58] and participates in the process of information processing for touch and pain [59, 60]. Theta oscillation also plays a crucial network-level role in hippocampal learning and memory; thus, suppression of the theta rhythm impairs learning and memory [61]. These findings support our understanding for the role of low-frequency oscillations in pain processing [62].

Moreover, changes in wide-range band oscillations have been seen in several acute and chronic pain models. Under noxious stimulation, decreased power of alpha and beta bands and increased power of the gamma band have been reported [63, 64]. In a separate study, the authors found that the power of delta, gamma, and epsilon bands was generally increased whereas the power of alpha and beta bands was decreased in rats that received noxious laser heat stimulation [65]. In neuropathic rats, acute mechanical pain stimulation leads to increased alpha and decreased beta and gamma oscillations in the medial prefrontal cortex (mPFC) LFP, while the delta oscillation is decreased and the gamma oscillation is dynamically decreased during chronic neuropathic pain condition [66]. Additionally, in freely moving rats with carrageenan-induced inflammation, application of von Frey mechanical stimulation to the ipsilateral paw results in a significant increase in delta, theta, and alpha bands of LFP in the ACC, suggesting that there are significant changes in the low-frequency oscillations of LFP occurred during inflammatory pain [67]. So, an increased theta band oscillation of VPL LFP in RF-treated rats may suggest the role of low-frequency oscillation, particularly in the VPL, in RF-induced hyperalgesia.

In addition, cross-frequency coupling has functional significance in cortical information processing, thereby contributing to the integration of relevant regions in the brain [68]. In human beings, painful laser stimulation enhance gamma oscillatory responses in the pain-related amygdala and hippocampal regions, and these gamma responses are significantly coupled with the phases of theta and alpha rhythms during pain processing [69]. Painful cutaneous laser stimuli also induce event-related gamma band activity in the lateral thalamus in humans, and thalamic cross-frequency coupling analysis indicates that the phase of the lower frequency activity (theta to beta) modulates the amplitude of the higher frequency activity (low and high gamma) more strongly during the cutaneous laser stimuli [70]. In rats with visceral pain, an increased theta band power is observed in the ACC, and the neuronal spike activity in the ACC becomes synchronized with ongoing theta oscillations of LFP [71]. Moreover, an enhanced synchronization of thalamo-ACC theta band LFPs was consistent with an increased neuronal communication between the two regions [71]. These results reveal that theta band oscillations and theta frequency phase-locking may serve as prominent features of neuronal activity in the ACC and a potential neural mechanism underlying acute visceral pain. Consistently, our present data showed that the activity of the theta band oscillation in VPL LFP was significantly increased on day 3 post-RF infusion. These data suggest that the VPL may promote the development of RF-induced hyperalgesia by regulating the pain-related cortical areas.

It is accepted that thalamocortical oscillations are critical for sensory perception [72], and pain is known to disrupt synchrony in thalamocortical oscillations. Thalamocortical coherence has been reported to be increased in patients with neurogenic pain [42] but decreased in a rat model of central pain [73]. In a separate study, the authors also reported that the thalamocortical coherence between VPL and SI is decreased while the theta power amplitude in the thalamus and cortex is increased in rat models of acute and chronic pain [74]. It has been suggested that the thalamus regulates and coordinates with the cerebral cortex through direct anatomical connections rather than distal synapses and that the low-frequency theta rhythm in the thalamus can directly regulate high-frequency cortical activity in the beta or higher range [75, 76]. Also, the clinical studies have found that the power of the theta band in the VPL was negatively correlated with pain relief; that is, the power of the theta band was increased during the onset of pain [8]. In agreement with these findings, our present results showed that the power spectral density (PSD) in the theta band of VPL LFP was significantly increased after RF infusion, which support our hypothesis that the VPL may promote the development of RF-induced hyperalgesia by coupling with high-frequency oscillatory activity in other pain-related cortical areas.

### 6.4. The Analgesic Effects of EA on the Remifentanil-Induced Hyperalgesia in Rats

The frequency is one of the important parameters for EA treatment. Low-frequency (2 Hz) EA was effective for the treatment of inflammatory pain and neuropathic pain [13, 77], while high-frequency (100 Hz) EA was better for the spinal injury-induced muscle crampy pain [78]. In this study, we validated these findings by showing that 2 Hz-EA indeed has a therapeutic effect on RF-induced hyperalgesia in rats. Additionally, we found that the current intensity is another key parameter for EA treatment. Several studies have shown that the low-intensity (1 mA) EA is superior to the high-intensity (5 mA) EA for the analgesic effect of 2 Hz-EA on the CCI-induced neuropathic pain. For patients with postoperative pain, transcutaneous electrical acupoint stimulation (TEAS) with strong current intensity is more effective [79]. We have tested the analgesic effect of 2 Hz-EA on RF-induced hyperalgesia with low-intensity (0.5–1.5 mA) and high-intensity (1–3 mA) EA in our preliminary experiments and found that the high-intensity EA could effectively alleviate the RF-induced hyperalgesia. Moreover, acupoints selection is also an important factor influencing the efficacy of EA treatment. *Zusanli* (ST.36) and *Sanyinjiao* (SP.6) are usually applied to produce the analgesic effect [14]. In this study, we found that 2 Hz-EA had a significant analgesic effect on the RF-induced hyperalgesia in rats by stimulating bilateral ST.36 and SP.6 acupoints.

### 6.5. Mechanisms Underlying the Analgesic Effects of EA on the Remifentanil-Induced Hyperalgesia

It is reported that EA treatment inhibits the activity of nociceptive neurons in the VPL [19]. Studies have found that 94% of the VPL neurons can be activated by innoxious and noxious stimuli in peripheral receptive fields, while 6% of the VPL neurons only respond to noxious stimuli, and there is no neuron in the VPL that only respond to innoxious stimuli [80]. In line with these findings, we discovered that 2 Hz-EA can significantly inhibit the increased spontaneous firing rate of VPL neurons in rats with RF-induced hyperalgesia. We thus assumed that these inhibitory effects of 2 Hz-EA on spontaneous activity of VPL neurons should be, at least in part, responsible for the analgesic effects of EA on RF-induced hyperalgesia.

Analysis of LFP in the VPL showed that 2 Hz-EA also abrogates the increased power spectral density (PSD) of the theta band LFP in the VPL in RF-treated rats. Our results are consistent with previous findings showing that the low-frequency (2–10 Hz) stimulation of the thalamus reduces the theta band oscillation of the cortex and the nociceptive behaviours [81–83]. As mentioned above, thalamocortical oscillations are critical for sensory perception [72] and that the low-frequency theta rhythm in the thalamus can directly regulate high-frequency cortical activity in the beta, gamma, or higher ranges [75, 76]. Therefore, coupling between theta and gamma is involved in various acute and chronic pain processing [66, 71]. On the other hand, some clinical and preclinical studies have demonstrated that the power of the theta band oscillations in the VPL is negatively correlated with pain relief and EA at 2 Hz frequency reverses the increased beta power and the cross-frequency couplings between beta and low-frequency theta band oscillations induced by postincisional pain [8], implying that EA regulates the neuronal networks involved in the central processing and in the integration of spontaneous nociceptive information. Together these findings with our present results, we speculated that EA may serve as an “antagonist” for theta band oscillation of VPL LFP and be able to disrupt the cross-frequency couplings between low-frequency theta band and high-frequency cortical activities. Thus, the analgesic effects of 2 Hz-EA on the RF-induced hyperalgesia is likely mediated by suppressing the low-frequency (theta band) oscillation of LFP in the VPL.

## 7. Conclusions

In summary, our present results demonstrated that a 2-hour remifentanil infusion induces a substantial pain hypersensitivity in rats and also enhances the spontaneous neuronal firing rate and the activity of the theta band LFP in the VPL. 2 Hz-EA attenuates the remifentanil-induced hyperalgesia via inhibiting the excitability of VPL neurons and the activity of low-frequency theta band oscillation in VPL LFP. The analgesic effects of EA on RF-induced hyperalgesia through its "antagonistic" role in theta band oscillation of VPL LFP also provides an extra evidence for the contribution of neural oscillation to the pathogenesis of OIH.

## Data Availability

The data used to support the findings of this study are available from the corresponding author upon request.
